# Modelling and Optimization of Four-Segment Shielding Coils of Current Transformers

**DOI:** 10.3390/s17061218

**Published:** 2017-05-26

**Authors:** Yucheng Gao, Wei Zhao, Qing Wang, Kaifeng Qu, He Li, Haiming Shao, Songling Huang

**Affiliations:** 1 Department of Electrical Engineering, Tsinghua University, Beijing 100084, China; yuchengg@gmail.com (Y.G.); zhaowei@tsinghua.edu.cn (W.Z.); huangsling@tsinghua.edu.cn (S.H.); 2 School of Engineering and Computing Sciences, Durham University, Durham DH1 3LE, UK; 3 Beijing Internet Based Engineering Co., Ltd., Beijing 100083, China; kf.qu@iadiae.com; 4 Institute of Instrumentation and Measurement, State Grid Electric Power Research Institute, Wuhan 430074, China; lihe3@epri.sgcc.com.cn; 5 Division of Electricity and Magnetism, National Institute of Metrology, Beijing 100029, China; shaohm@nim.ac.cn

**Keywords:** current transformers, current measurement, electromagnetic interference, magnetic flux leakage, magnetic shielding

## Abstract

Applying shielding coils is a practical way to protect current transformers (CTs) for large-capacity generators from the intensive magnetic interference produced by adjacent bus-bars. The aim of this study is to build a simple analytical model for the shielding coils, from which the optimization of the shielding coils can be calculated effectively. Based on an existing stray flux model, a new analytical model for the leakage flux of partial coils is presented, and finite element method-based simulations are carried out to develop empirical equations for the core-pickup factors of the models. Using the flux models, a model of the common four-segment shielding coils is derived. Furthermore, a theoretical analysis is carried out on the optimal performance of the four-segment shielding coils in a typical six-bus-bars scenario. It turns out that the “all parallel” shielding coils with a 45° starting position have the best shielding performance, whereas the “separated loop” shielding coils with a 0° starting position feature the lowest heating value. Physical experiments were performed, which verified all the models and the conclusions proposed in the paper. In addition, for shielding coils with other than the four-segment configuration, the analysis process will generally be the same.

## 1. Introduction

Magnetic interference is a serious problem for the heavy current transformers (CTs) installed at the end terminal of large capacity generators [1,2,3,4,5]. The main component of this interference is the stray flux produced by the adjacent bus-bars (hereafter referred to as stray flux). Because of the distance limitations between the multiphase bus-bars (about 1 m) and the magnitude of the bus-bar current (about 5–40 kA), the stray flux is strong enough to cause a partial magnetic saturation in the current transformer (CT) ring core, consequently leading to measurement error and even permanent damage in severe cases [6,7].

**Figure 1:**
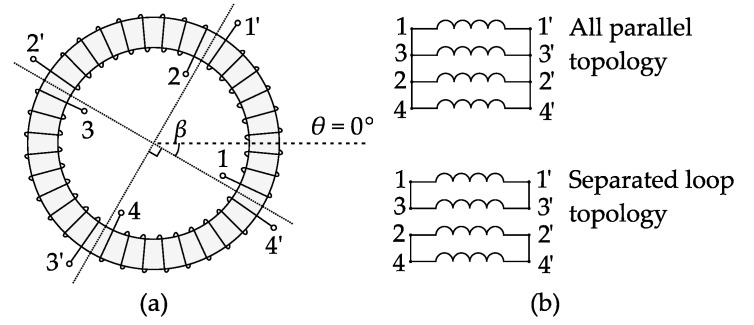
Diagrams of four-segment shielding coils for CT: (**a**) Structural diagram; (**b**) Connecting topologies.

Several solutions are available to shield the stray flux, such as that proposed in [8]. Another common solution to shield the stray flux is to introduce shielding coils [2,9] (also known as flux equalizing windings [10,11]) on the CT iron core. Under the alternating stray flux, the shielding coils will generate a leakage flux (hereafter referred to as coil leakage flux) that neutralizes the stray flux [12], and thereby avoids core saturation. There are several types of shielding coil, and the four-segment type, illustrated in Figure 1, is among the most commonly used [10,13]. The coils are wound over the secondary winding. The four coil segments can be connected in either “separated loop topology” or “all parallel topology”, as shown in Figure 1b. Theoretically, there is no limit to the coils’ starting position *β*.

However, so far, quantified studies on the performance of shielding coils are limited in number and scope. Due to the lack of analytical models, existing studies on the stray flux and the leakage flux of shielding coils are mainly based on physical experiments [1,4,10,13,14,15] or numerical calculations [9,11,13,14,16,17,18], both of which take a lot of time to process [19]. This problem is especially pronounced in the optimization of shielding coils, where thousands of cases are to be inspected [20]. Moreover, previous studies have focused on the interfering stray flux of the single adjacent bus-bar, and whether the conclusions can be generalized to practical cases with multiphase bus-bars has not yet been verified.

An analytical model of the interfering flux will be an effective solution to this problem. For the stray flux, there have been several attempts at building such models. The first analytical equation of the stray flux was deduced through an equivalent magnetic circuit method [1]. However, there is an empirical factor in the equation, and since the study in [1] was carried out on small-size current transformers, the empirical factor graph proposed in [1] is not applicable to mainstream CTs for large capacity generators. In recent years, another stray flux equation has been derived using the magnetic vector potential (MVP) method [16], and this equation is essentially the same as the equivalent magnetic circuit-based equation, suggesting that it is universally applicable to ring cores of all sizes. Nevertheless, as a proper method to determine the empirical factor is still unavailable, the equation is not capable of calculating the stray flux accurately.

For the coil leakage flux, an analytical model of coil leakage flux for CTs has not yet been introduced. Instead, a finding was demonstrated in [14] that the coil leakage flux is equivalent to the stray flux under certain conditions. Still, the equivalence is rather rough and not accurate enough to be used for quantified studies, especially for solving the coil currents based on the leakage flux.

The objective of this paper is to build and improve analytical models for the stray flux and the coil leakage flux, so that analysis and optimization of shielding coils can be performed effectively and conveniently. Firstly, the stray flux model proposed in [1] is improved, and a coil leakage flux model is introduced. Secondly, the common four-segment shielding coils are taken as an example to show how the shielding coil model is built. Thirdly, the optimization of the four-segment shielding coils is presented as an application example of the analytical models. Finally, the design and the results of the verifying experiments are discussed in detail.

## 2. Analytical Models of the Stray Flux and Coil Leakage Flux

**Figure 2:**
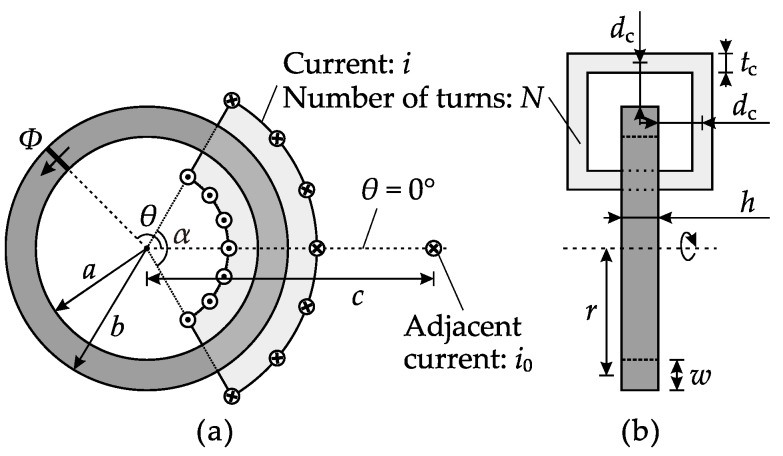
Cross-sectional views of the CT ring core: (**a**) Front view; (**b**) Top view.

A front cross-sectional view and a top cross-sectional view of a CT ring core are shown in Figure 2, where the dark region is the core, and the light gray areas represent the coil. A cylindrical coordinate system is used in this model. The origin is located at the centroid of the core, and the *z*-axis coincides with the axis of the core. Let the position of the adjacent bus-bar, or the midpoint of the coil, be *θ* = 0°.

The following assumptions are made to simplify the analysis. In operating conditions, the eligible shielding coils will keep the magnetic field in the CT ring core at a low intensity so that magnetic saturation will not occur. Consequently, a linear model is applicable for the analysis, and the relative permeability of the ring core is considered as a constant far larger than 1. In addition, the influence of eddy currents and the hysteresis effect are also neglected in the model.

### 2.1. Stray Flux Model

A diagram of the cross-sectional views of the CT ring core is presented in Figure 2, which also explains all the variables in the model. According to [1], in the cross-section area of angle *θ* (the bold line in the front view), the stray flux produced by an adjacent bus-bar is

(1) $$\Phi_{s}\left(\theta \right)=\frac{\mu_{0}k_{s}hi_{0}}{\pi }\sum_{k=1}^{\infty }\frac{b^{n}}{n\cdot c^{n}}\cos\left(n\theta \right)$$

where *μ*_0_ is the permeability constant, and *k*_s_ is the core-pickup factor for the stray flux, which is determined by an empirical plot proposed in [1]. All the other symbols are defined in Figure 2.

Equation (1) can also be derived from the vector potential method, suggesting that it is a universal equation that applies to ring-type iron cores of all sizes. The only problem, however, is the evaluation of *k*_s_—the empirical plot is not applicable to the currently used mainstream heavy CTs because the typical dimensional range of the mainstream CTs, as shown in Table 1, is notably different to that of the CTs discussed in [1].

To propose a new empirical equation for the currently used mainstream CTs, sufficient empirical data are required. The empirical data can be obtained by numerical simulation based on the finite-element method (FEM), a method that has been proven to be highly accurate in solving stray flux in a CT core [17].

In this study, FEM-based simulations were implemented using commercial software, ANSYS Workbench 15.0, developed by Ansys Inc., Canonsburg, PA, USA. The simulation mode was set to magnetostatic analysis. In order to model this open boundary problem, a large cylindrical air region was built, with the ring core object horizontally placed at the center of the region. The radius and the height of the cylindrical air region were respectively set to 4 m and 5 m, which had been validated to be accurate enough for this analysis [17]. The ring core object was meshed into approximate 10 × 10 × 10 mm cubic elements, and the air region was meshed freely in default parameters. Furthermore, bus bars with a 50 × 50 mm square cross section were used as the primary and adjacent bus bars. This design is a compromise between the mesh size and the assumptions of the analytical model.

The core-pickup factor *k*_s_ is defined as the solution of the following optimization problem about *k*

(2) $$\underset{k}{\min}\left\|\Phi_{s-\mathrm{sim}}\left(\theta \right)-k\cdot \frac{\mu_{0}hi_{0}}{\pi }\sum_{n=1}^{\infty }\frac{b^{n}}{k\cdot c^{n}}\cos\left(n\theta \right)\right\|$$

where *θ* varies from 0° to 360°, *Φ*_s-sim_(*θ*) is the simulation result of the stray flux, and the latter fraction is the analytical calculation result of the stray flux, as demonstrated in Equation (1).

Basically, there are four parameters that may affect the value of *k*_s_: *r*, *w*, *h*, and *c*. Accordingly, based on the control variable method, four simulation cases are designed and implemented, and the specifications of the cases are shown in Table 2.

**Figure 3:**
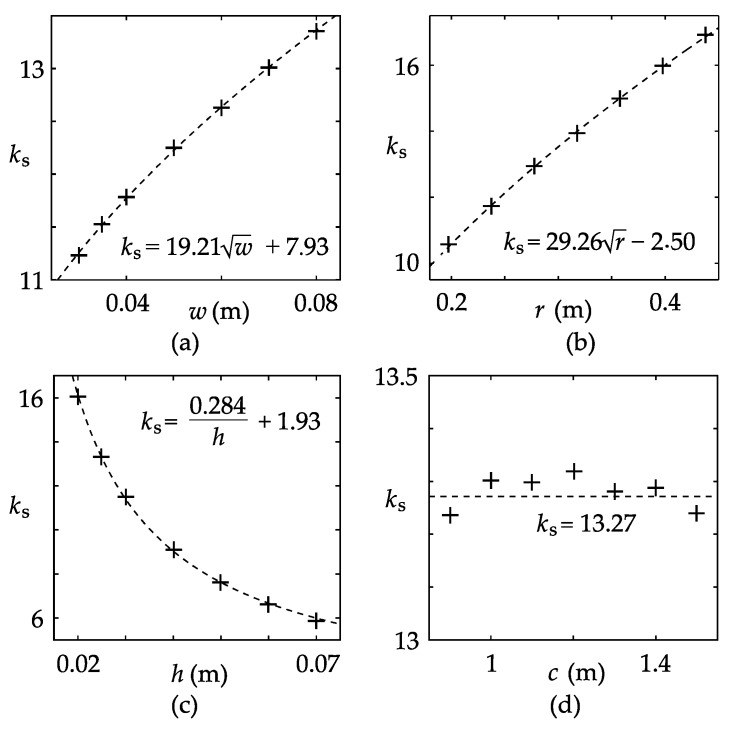
Plots of the core-pickup factor versus model parameters. The cross marks represent the simulation values, and the dashed lines are the curves of the fitting functions: (**a**) Case 1; (**b**) Case 2; (**c**) Case 3; (**d**) Case 4.

Figure 3 shows the simulation results of *k*_s_, along with the fitting curves of the results. The fitting functions are also annotated in the plots. Referring to Figure 3a–c, *k*_s_ is approximately linear to the square root of *r*, the square root of *w*, and the reciprocal of *h*, respectively. The correlation between *k*_s_ and *c* is very weak, according to Figure 3d, which means that *k*_s_ is irrelevant to the adjacent bus-bar and is entirely dependent on the CT core diameters. Thereby, the basic form of the empirical equation should be

(3) $$\begin{array}{cc} k_{s}\left(w,r,h\right) & =p_{1}\frac{\sqrt{wr}}{h}+p_{2}\frac{\sqrt{r}}{h}+p_{3}\frac{\sqrt{w}}{h}+p_{4}\frac{1}{h}\\ & +p_{5}\sqrt{wr}+p_{6}\sqrt{r}+p_{7}\sqrt{w}+p_{8} \end{array}$$

where *p*_1_–*p*_8_ are constant coefficients. To determine these coefficients, a total of 180 simulation cases were carried out, where *r* = 0.20, 0.25, 0.30, 0.35, 0.40 m; *w* = 0.02, 0.03, 0.04, 0.05, 0.06, 0.07 m; *h* = 0.02, 0.025, 0.03, 0.04, 0.05, 0.06 m; and *c* = 1.2 m. Based on the simulation results, the coefficients were obtained through fitting analysis, as listed in Table 3. The mean squared error (MSE) of the fitting analysis is 0.091, which is approximately 0.5–1% of *k*_s_.

Furthermore, in [16], the leakage flux produced by an eccentric primary bus-bar is also derived from the vector potential method, as shown in Equation (4).

(4) $$\Phi_{e}\left(\theta \right)=\frac{\mu_{0}k_{e}hi_{1}}{\pi }\sum_{k=1}^{\infty }\frac{d^{k}}{k\cdot a^{k}}\cos\left(k\theta \right)$$

where *d* is the eccentric distance of the bus-bar, *i*_1_ is the bus-bar current, and *k*_e_ is the core-pickup factor for the leakage flux produced by the eccentric bus-bar, which has been estimated to be almost the same as *k*_s_ by simulation and experimental results demonstrated in previous publications [15,17].

### 2.2. Coil Leakage Flux Model

A one-turn coil on a CT core is the combination of four conductors, two of which are placed in the *z* direction and the other two which are placed in the *r* direction, as shown in Figure 2b. To simplify the model, the distance between the CT surface and the center of the conductor is set to *d*_c_ for all four conductors, which is the most common case for shielding coils. In the 2D model shown in Figure 2a, the one-turn coil is further simplified into an adjacent conductor and an eccentric primary conductor, whereas the effect of the *r* direction conductors is calibrated by a core-pickup factor, which will be discussed later. Therefore, the leakage flux produced by the one-turn coil is the sum of the stray flux of the adjacent current and the leakage flux of the eccentric current, where *c* = *b* + *d*_c_, *d* = *a* − *d*_c_. Since *a* and *b* are usually similar and far larger than *d*_c_, a mathematical approximation is practicable, which is

(5) $$\frac{a-d_{c}}{a}\approx \frac{b}{b+d_{c}}$$

Combining Equations (1) and (4), the leakage flux of the single turn of the electrified coil is shown in Equation (6).

(6) $$\Phi_{c1}\left(\theta \right)=\frac{\mu_{0}k_{c}hi}{\pi }\sum_{n=1}^{\infty }\frac{2b^{n}}{n\cdot {\left(b+d_{c}\right)}^{n}}\cos\left(n\theta \right)$$

In Equation (6), *k*_c_ is the core-pickup factor for the coil leakage flux equation, which is not completely equal to *k*_s_, because a one-turn coil is not equivalent to a pair of bus-bars in the 3D model. To describe the difference between the two core-pickup factors, a new factor *k*_cs_ is introduced, as defined in Equation (7).

(7) $$k_{\mathrm{cs}}=\frac{k_{c}}{k_{s}}$$

The leakage flux of a coil on CT (*Φ*_c_) is the sum of *Φ*_c1_ of each turn. As long as the number of turns is large, which means the coil density is sufficiently high, the leakage flux of the coil is equal to the integral of *Φ*_c1_ from the start angle (−*α*/2) to the end angle (*α*/2) of the coil.

(8) $$\Phi_{c}\left(\theta \right)=\frac{4\mu_{0}hk_{s}k_{\mathrm{cs}}Ni}{\pi \alpha }\sum_{n=1}^{\infty }\frac{b^{n}\sin\left(n\alpha /2\right)}{{\left(b+d_{c}\right)}^{n}n^{2}}\cos\left(n\theta \right)$$

A FEM-based simulation was again implemented to find the empirical equation of *k*_cs_. The coil objects were meshed into approximate 10 × 10 × 10 mm cubic elements, and all the other simulation settings remained the same. Three practical CTs were used in the simulation, as listed in Table 4.

Basically, there are three parameters that may affect the value of *k*_cs_: *α*, *d*_c_, and *t*_c_. Accordingly, based on the control variable method, cases 5–7 were designed, as follows:

- Case 5: change *d*_c_ from 0.025 m to 0.15 m (30 kA and 25 kA CTs), or from 0.0175 m to 0.1 m (5 kA CT), while keeping *α* and *t*_c_ as default values;
- Case 6: change *α* from 75° to 150° stepped by 15°, while keeping *d*_c_ and *t*_c_ as default values;
- Case 7: change *t*_c_ from 0.01 m to 0.05 m stepped by 0.01 m, while keeping *α* and *d*_c_ as default values.

**Figure 4:**
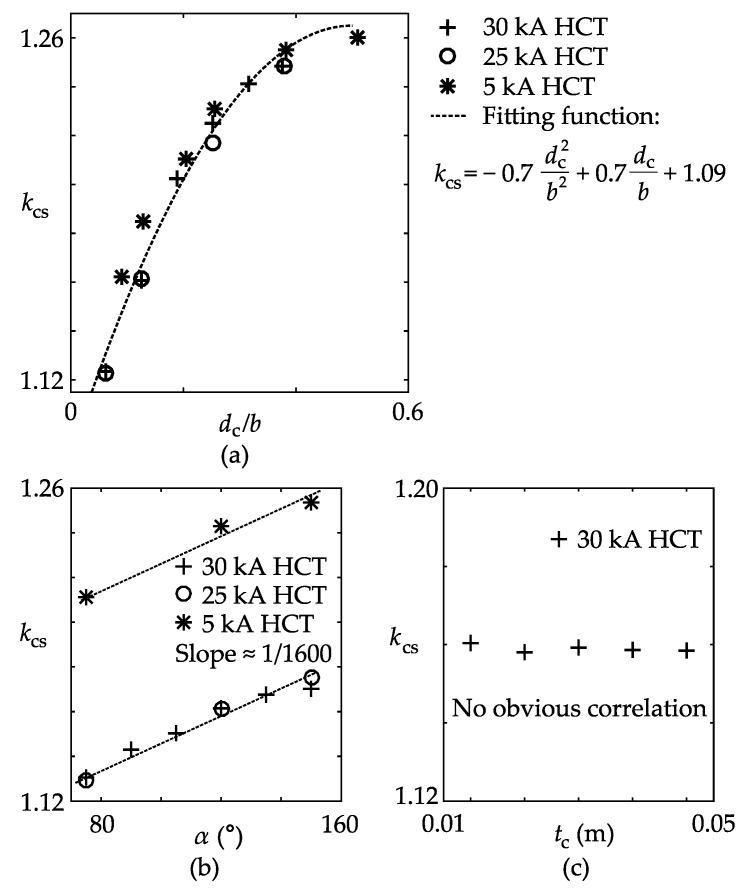
Plots of the core-pickup factor versus model parameters: (**a**) Case 5; (**b**) Case 6; (**c**) Case 7.

The simulation result of *k*_cs_ is plotted in Figure 4. In Figure 4a, the variation of *k*_cs_ is observed to be quadratic, and the three curves nearly coincide with each other, suggesting that *k*_cs_ is strongly dependent on the ratio *d*_c_/*b*. The curves in Figure 4b are approximately parallel lines, implying that *k*_cs_ is a linear function of *α*, and the slope is irrelevant to the other parameters. In Figure 4c, only the result of the 30 kA CT is presented, which is almost a horizontal line, proving that *k*_cs_ is not influenced by *t*_c_.

After performing fitting analysis, the following approximate equation of *k*_cs_ was concluded, as shown in Equation (9).

(9) $$k_{\mathrm{cs}}=-0.7{\left(\frac{d_{c}}{b}\right)}^{2}+0.7\frac{d_{c}}{b}+\frac{\alpha }{1600{}^{\circ}}+0.015$$

With this, the coil leakage flux model is constructed. Using Equations (3), (8) and (9), the leakage flux of any coil on a mainstream CT core can be calculated.

## 3. Modeling of Four-Segment Shielding Coils

In this section, a model of the four-segment shielding coils (presented in Figure 1) is established. For other kinds of shielding coils, the idea of modeling will be generally the same. A FEM-based simulation verification and an experimental verification of this model will be given in Section 5.

**Figure 5:**
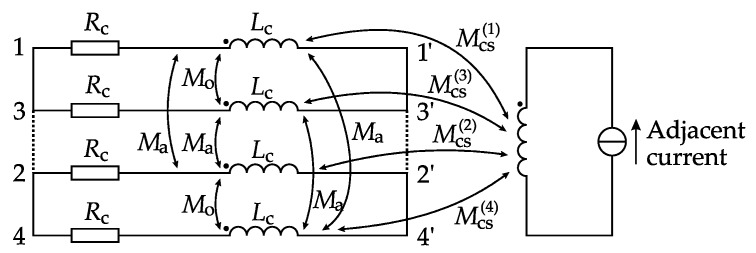
Circuit diagram of the shielding coils. The dashed lines are connected only in “all parallel topology”.

The aim of the shielding coil model is to find the coil currents, as well as the in-core remnant flux when the shielding coils are energized. One of the most convenient ways to calculate currents is to simplify the electromagnetic model as an electrical circuit model. In this study, each shielding coil can be modeled as a series circuit consisting of an internal resistance, a self-inductance, and mutual inductances between the coil and another coil or the adjacent bus-bar [21]. A circuit diagram of the shielding coils is shown in Figure 5.

As the internal resistance of the coils is small and hence negligible, the key to the model is to accurately determine all the inductances in the circuit. According to the definition, inductance is the ratio of flux linkage to current, and the flux linkage is proportional to flux. The flux produced by each shielding coil can be divided into two parts: the leakage flux, which is described by a function *Φ*_c_(*θ*) defined in Equation (8); and the main flux, which is universal in the core and is described by a constant *Φ*_cm_. It will be explained later that *Φ*_cm_ does not need to be solved in this model.

To simplify the calculation, only the flux linkage passing through the core is taken into consideration. As the number of coil turns *N*_c_ is high, the flux linkage can be regarded as the integration of the flux over *θ*. Therefore, the self-inductance of each shielding coil is

(10) $$L_{c}=\frac{2N_{c}}{\pi i_{c}}\int_{-\frac{\pi }{4}}^{\frac{\pi }{4}}\left[\Phi_{c}\left(\theta \right)+\Phi_{\mathrm{cm}}\right]d\theta =L_{\mathrm{cm}}+\sum_{n=1}^{\infty }p_{n}\sin\frac{n\pi }{4}$$

where

(11) $$p_{n}=\frac{32\mu_{0}k_{s}k_{\mathrm{cs}}N_{c}^{2}}{\pi^{3}}\cdot \frac{b^{n}}{{\left(b+d_{c}\right)}^{n}n^{3}}\sin^{2}\left(\frac{n\pi }{4}\right)$$

(12) $$L_{\mathrm{cm}}=\frac{N_{c}\Phi_{\mathrm{cm}}}{i_{c}}$$

The mutual inductance between adjacent segments of the shielding coils is

(13) $$M_{a}=\frac{2N_{c}}{\pi i_{c}}\int_{\frac{\pi }{4}}^{\frac{3\pi }{4}}\left[\Phi_{c}\left(\theta \right)+\Phi_{\mathrm{cm}}\right]d\theta =L_{\mathrm{cm}}+\sum_{n=1}^{\infty }p_{n}\cos\frac{n\pi }{2}$$

The mutual inductance between opposite segments of the shielding coils is

(14) $$M_{o}=\frac{2N_{c}}{\pi i_{c}}\int_{\frac{3\pi }{4}}^{\frac{5\pi }{4}}\left[\Phi_{c}\left(\theta \right)+\Phi_{\mathrm{cm}}\right]d\theta =L_{\mathrm{cm}}+\sum_{n=1}^{\infty }p_{n}\cos n\pi$$

The mutual inductances between the adjacent bus-bar and segment *q–q*′ (*q* = 1, 2, 3, 4) of the shielding coil are

(15) $$M_{\mathrm{cs}}^{\left(q\right)}=\frac{2\mu_{0}k_{s}h}{\pi^{2}}\sum_{n=1}^{\infty }\frac{b^{n}}{n^{2}c^{n}}\sin\frac{n\pi }{4}\cos\left(n\beta -\frac{\left(2q-1\right)n\pi }{4}\right)$$

In addition, in either “all parallel topology” or “separated loop topology”, the following equation is constantly satisfied.

(16) $$i_{1}+i_{2}+i_{3}+i_{4}=0$$

Therefore, in the voltage equation of each branch of the circuit, the sum of the terms containing *L*_cm_ is constantly zero. As a result, *L*_cm_ is irrelevant to the coil currents and is thus ignored in the model. At this point, all the parameters in the circuit are determined, and the coil currents can then be solved.

Next, the remnant flux in the core can also be solved, which is the sum of the stray flux, the coil leakage flux produced by all the balance coils, and the main flux that is universal in the core. In normal conditions, the primary and adjacent currents are sinusoidal, and the remnant flux can be expressed in phasor form, as shown in Equation (17).

(17) $${\dot{\Phi }}_{r}\left(\theta \right)={\dot{\Phi }}_{s}\left(\theta \right)+\sum_{q}{\dot{\Phi }}_{cq}\left(\theta \right)+{\dot{\Phi }}_{m}$$

where ${\dot{\Phi }}_{cq}\left(\theta \right)$ is the coil leakage flux phasor of the balance coil *q–q*′, ${\dot{\Phi }}_{s}\left(\theta \right)$ is the stray flux, and ${\dot{\Phi }}_{m}$ is the main flux phasor, which leads about 90° ahead of the secondary current. The approximation of the main flux phasor is

(18) $${\dot{\Phi }}_{m}=-j\frac{\sqrt{2}{\dot{E}}_{2}}{2\pi f}=-j\frac{R_{2}}{4.44fN_{2}}{\dot{I}}_{2}$$

where *İ*_2_, *R*_2_, *N*_2_, and *f* are the secondary current phasor, the resistance of the secondary winding, the number of turns of the secondary winding, and the frequency of the current, respectively.

## 4. Optimization of the Four-Segment Shielding Coils

To implement a quantitative analysis of the shielding performance, the following two quantities are introduced as performance parameters:

- |*Φ*_r_|_max_—the maximum absolute value of *Φ*_r_. When the dimension of the core is certain, |*Φ*_r_|_max_ is approximately proportional to the peak flux density of the remnant magnetic field. If |*Φ*_r_|_max_ is too high, the core will be in danger of saturation.
- $I_{b-\max}^{2}$—the square of the max *I*_b_ of the four coils. When the resistance of the balancing coils is fixed, $I_{b-\max}^{2}$ is proportional to the heat produced by the shielding coils. If $I_{b-\max}^{2}$ is too high, the CT will be in danger of overheating.

**Figure 6:**
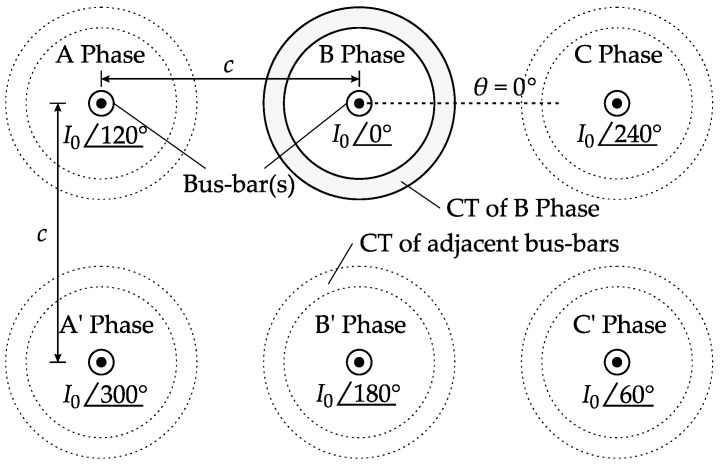
Layout diagram of the six-phase bus-bars and B phase CT.

A calculation example was implemented to evaluate how *β* and connection topology influence the shielding performance. The example used a 30 kA generator CT, whose parameters are given in Table 5. In the example scenario, the generator terminal had six bus-bars, attributed to the three phases and their returning phases, as shown in Figure 6. The example CT was equipped on the B phase bus-bar, where the interfering magnetic field is believed to be the most intensive. The CT was interfered with by a total of five adjacent bus-bars.

According to the symmetry of the four coils, it can be inferred that as *β* increases, the calculation result repeats in a 90°-cycle. Therefore, in the example, the coil position *β* was changed from −45° to 45°. Meanwhile, both of the coil connection topologies were successively studied. |*Φ*_r_|_max_ and $I_{b-\max}^{2}$ were solved using the mathematical model proposed in Section 3.

**Figure 7:**
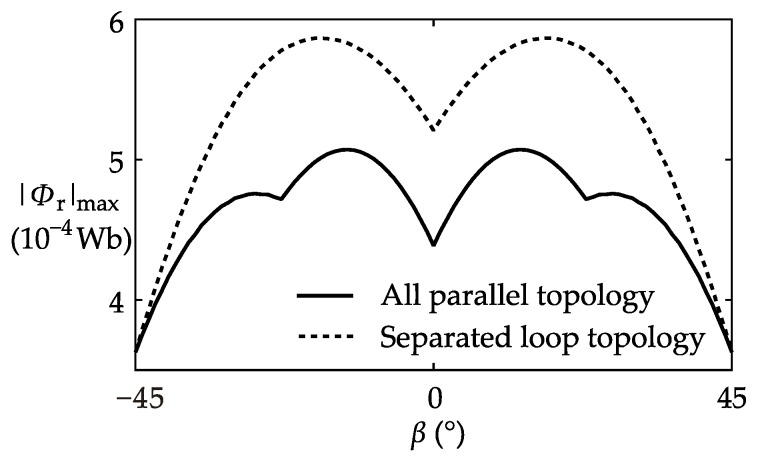
Plot of the calculation result of |*Φ*_r_|_max_.

**Figure 8:**
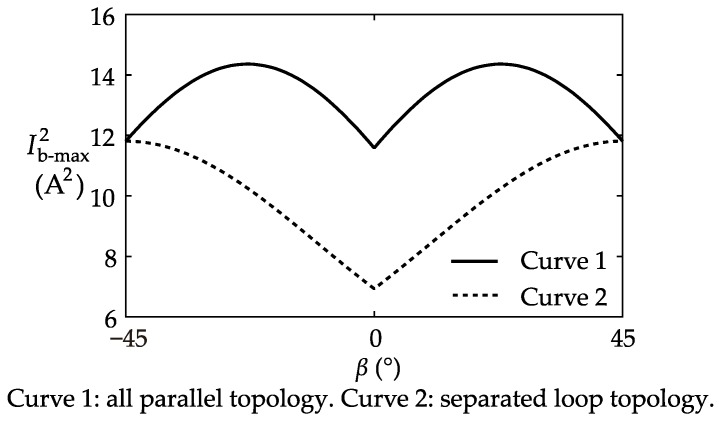
Plot of the calculation result of $I_{b-\max}^{2}$.

The results of the calculation example are plotted in Figure 7 and Figure 8. In the *β–*|*Φ*_r_|_max_ plot shown in Figure 7, both curves reach the same minimum value at *β* = 45°. However at other coil positions, the “all parallel topology” curve is always lower than the “separated loop topology” curve. The result signifies that according to the parameters of |*Φ*_r_|_max_, *β* = 45° is the optimal position, and the “all parallel topology” is the better connection topology.

In the *β–*$I_{b-\max}^{2}$ plot of Figure 8, the curve of the “separated loop topology” shielding coils has a significantly lower minimum point, which is located at *β* = 0°. In the global scope, the “separated loop topology” curve is always below the other curve. From the results, it is concluded that according to $I_{b-\max}^{2}$, the “separated loop topology” is the superior connection topology, and *β* = 0° is the best coil position.

The two plots lead to completely opposite optimal solutions of the shielding coils. In other words, if stray fields are better compensated, a larger shielding coil current is necessary, and inevitably more heat is generated that may affect the CT. Therefore, when designing the shielding coils of a practical CT, it needs to be decided which performance parameter should be treated as preferential. As the heating of the shielding coils can be reduced by increasing the number of turns of the coils, it is usually the top priority to reduce |*Φ*_r_|_max_, and, as a result, the *β* = 45° “all parallel topology” shielding coils will be selected. However, if the interfering field is not very intensive, the *β* = 0° “separated loop topology” shielding coils can be applied to reduce the usage of copper for the shielding coils.

## 5. Examples

### 5.1. Verification of the Analytical Models

To verify the analytical models proposed in Section 2 and Section 3, an experiment was carried out on a 30 kA CT sample, whose parameters are shown in Table 5. A four-meter-long primary bus-bar was passed through the center of the CT ring. The returning conductor of the primary bus-bar was utilized as an interfering adjacent bus-bar, and the adjacent distance c was set to 1.2 m. The shielding coil position *β* was set to 0°. The secondary winding of the CT was shorted out by an ampere meter.

The main difficulties of the experiment are:

- Implementing a large sinusoidal current in the bus-bar;
- Powering the high-power circuit;
- Sustaining the large current for a few minutes.

**Figure 9:**
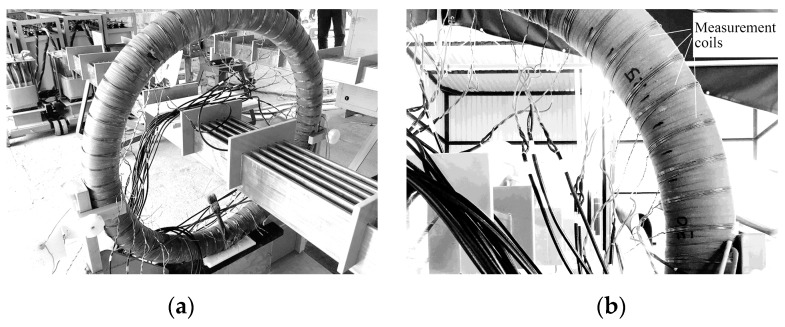
Workplace for the verification experiment of the analytical models. (**a**) 30 kA CT sample and primary bus-bar. (**b**) Pre-wound measurement coils.

To realize a large current, a six-turn bus-bar was used, as presented in Figure 9a. Therefore, the current in each copper bar is reduced to one-sixth of the total bus-bar current.

To lower the capacity requirement of the power source, reactive compensation was applied by connecting a 0.053 μF capacitor in series with the bus-bar. The whole circuit was connected to the secondary winding of a 380:8 transformer, and the primary winding of the transformer was powered by a 150 kVA, 0–220 V voltage regulator.

The sinusoidal flux in the CT core can be measured by enwinding a coil onto the CT and detecting the terminal voltage of the coil. However, as the voltage regulator cannot work under heavy-load conditions for long, the measurement should be taken quickly. To improve the measuring efficiency, a total of 30 uniformly distributed coils were enwound on the CT beforehand, as shown in Figure 9b. Every end of the coils was connected to a six-meter long wire, so the measurement of the coil voltage could be taken at a distance, preventing the strong magnetic field from influencing the voltmeters.

**Figure 10:**
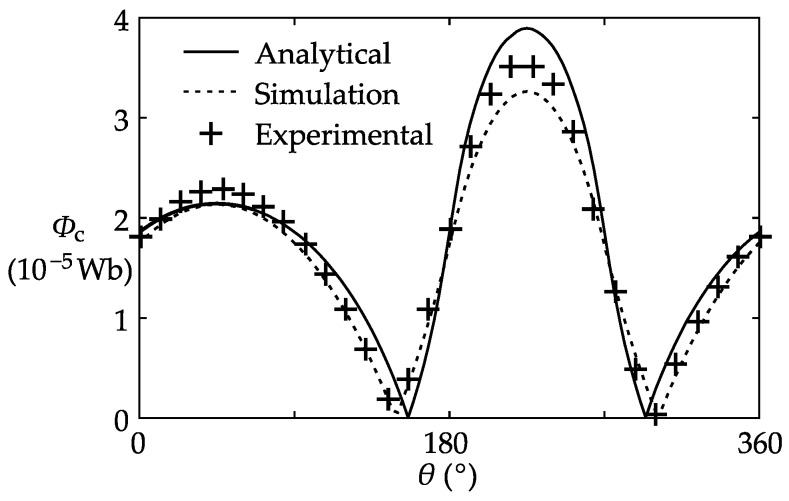
Analytical, simulation, and experimental results of the coil leakage flux.

The first step of the experiment was to verify the coil leakage flux model. At this step, the shielding coil 3–3’ (wound from *θ* = 180° to *θ* = 270°) was selected as an interfering coil and was provided with a 0.1 A AC current. Meanwhile, the other shielding coils and the bus-bar were not electrified. At this moment, the dominant flux in the core was the leakage flux produced by the shielding coil 3–3’. The flux measured in the experiment, along with the FEM simulation result and the analytical result of the coil leakage flux, are plotted together in Figure 10. It can be seen that the analytical curve coincides with the experimental curve, proving that the proposed coil leakage flux model defined by Equations (8) and (9) can accurately describe the leakage flux produced by a coil.

The next step was to verify the improved stray flux model. The bus-bar was powered with a 6000 ampere-turn AC current (1000 A for each copper bar), while all of the shielding coils were disconnected. When the circuit came into steady state, the flux in the core was approximately equal to the stray flux produced by the adjacent bus-bar.

**Figure 11:**
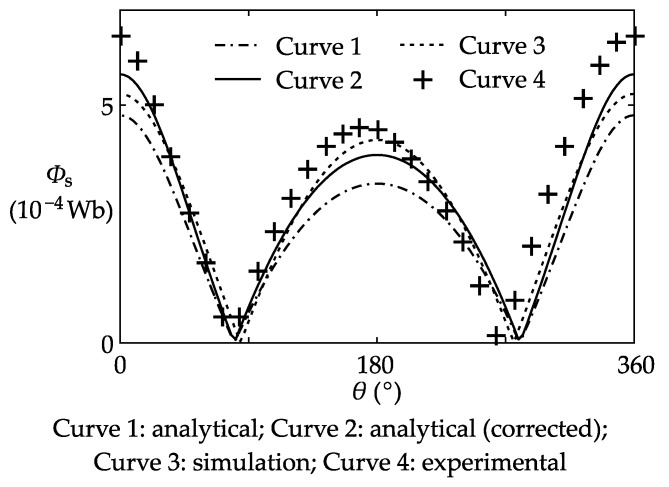
Analytical, simulation, and experimental results of the stray flux.

The experimental result, the FEM-based simulation result and the analytical result of the stray flux are plotted together in Figure 11. It was expected that with the correction factor *k*_s_, the simulation and the analytical curves would be close to each other. However, the analytical curve turned out to be slightly weaker. This discrepancy is due to the limited length (4 m) of the bus-bar in the model. The connecting bars between the primary and the adjacent bars, which are only 2 m away from the CT, intensifies the stray flux in the core. As a result, the flux in the simulation model is slightly stronger (about 18% in this case) than the analytical model, where the connecting bars are considered to be at infinity. When the analytical curve is corrected by a factor (about 1.18 in this case), it becomes close to the experimental curve, proving that the proposed stray flux model is capable of calculating the stray flux.

The last step was to verify the shielding coil model. The shielding coils were connected in the “all parallel topology”, and then the bus-bar current was finally raised to 24 kA (4000 A for each copper bar). The measured in-core remnant flux and the coil currents were recorded for analysis.

**Figure 12:**
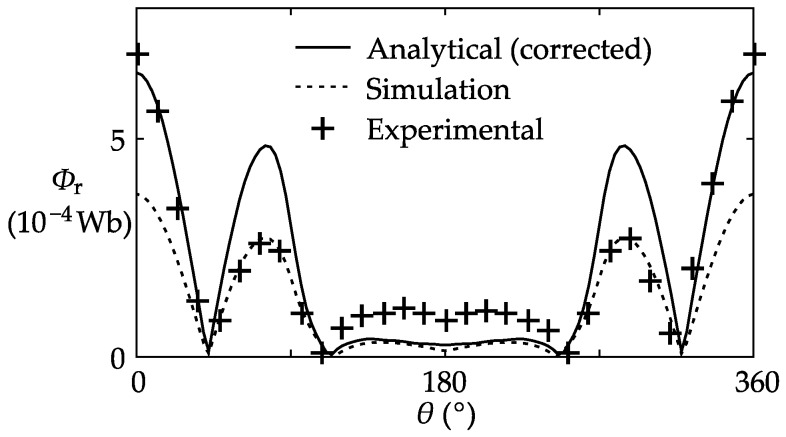
Analytical, simulation, and experimental results of the remnant flux.

The experimental results and the corrected analytical results of the remnant flux are plotted together in Figure 12. It can be seen that for both the remnant flux and the coil currents, the shape of the analytical curve is very close to the experimental curve, which validates the coil leakage flux model proposed in this paper. However, obvious disparities between the three curves can still be observed. It is worth noting that the remnant flux is the difference between two significantly larger quantities: the stray flux and the leakage flux produced by the shielding coils. Therefore, even if the discrepancies between the analytical, simulation, and experimental results are relatively small in the two intensive fluxes, the discrepancies will become much more significant in the remnant flux. Additionally, the figure shows that the experiment results are close to the analytical results when 0° < *θ* < 45°, and close to the simulation results when 45° < *θ* < 110°, but according to the analysis above, this is more likely to be a coincidence.

The results of the coil currents are listed in Table 6, showing that the analytical, simulation, and experimental results of the average coil currents are close to each other. In addition, the discrepancies between the currents of different segments are only observed in the experimental column. This is due to the inevitable deviations of the primary bus bar, the adjacent bus bar, and the shielding coils in the experimental setup. As the deviations are relatively small, they have very little influence on the flux and the average coil currents, but they do cause some extra circulating current and change the current distribution between the coils. However, they will not influence the general amplitude of the four coil currents.

### 5.2. Verification of the Optimal Shielding Coil Design

To verify the optimal shielding coil design for the three-phase scenario discussed in Section 4, a second experiment was carried out on a 5 kA CT sample, whose parameters are shown in Table 7. The 30 kA CT was substituted due to the difficulty in producing a set of three-phase 30 kA currents.

The experimental arrangement was similar to that of the first experiment. However, as the current in the three-phase experiment was lower, the number of turns of the bus-bar was reduced to 1. The adjacent distance *c* was set to 0.7 m, mirroring the case of a real 5 kA generator. A reactive compensation was applied by connecting a 3000 pF capacitor in parallel with the primary winding of the transformer. The arrangement was duplicated three times, to be the A, B, and C phases, respectively. The CT was installed on the B phase bus-bar, and the direction of the C phase bus-bar was defined as *θ* = 0°. As before, a total of 30 coils were enwound on the CT beforehand.

The shielding coil position *β* was set to 0° at first, and was then rotated to 45°. At each coil position, the shielding coils were connected in the “separated loop topology” and “all parallel topology” successively. Due to the limitation of the voltage regulators, the current of the bus-bars finally reached 4 kA.

The experimental and analytical results of the performance parameters are listed in Table 8. Although all of the experimental values are higher than the corresponding analytical values, the regulation of the performance parameters is identical to the theoretical analysis: the *β* = 0° shielding coils connected in the “separated loop topology” have the highest |*Φ*_r_|_max_ and the lowest $I_{b-\max}^{2}$; The *β* = 45° shielding coils connected in either way have the lowest |*Φ*_r_|_max_ and the highest $I_{b-\max}^{2}$.

The result proves the validity of the conclusions given in Section 4: setting *β* to 45° minimizes the remnant flux intensity, and the core can maximally avoid saturation; setting *β* to 0° and connecting the coils in the “separated loop topology” minimizes the temperature rise, and the CT is more unlikely to become overheated than in the other cases.

## 6. Conclusions

This paper has presented a practical way to calculate the interfering flux in CT iron cores. For the stray flux, an empirical equation was proposed to determine the core-pickup factor, which corrects the previous analytical model. The empirical equation is concluded based on a set of FEM-based simulations, and the mean squared error of the fitting equation is 0.071. For the leakage flux produced by the coils, a new analytical model was developed, and an empirical equation of the correction factor was also introduced. Based on the flux models, a model of the four-segment shielding coils was constructed. The interfering flux, the remnant flux, and the shielding coil currents calculated by the above models were compared to the ones obtained in the FEM-based simulations and physical experiments. It was shown that the calculation results are reasonably close to the simulation and experimental results, and with a remarkably shortened solving time.

These models were utilized to evaluate and optimize the performance of the shielding coils at an example generator terminal with a typical six-bus-bars layout. A parameter sweep was performed, whose results indicated that the “all parallel” shielding coils with a 45° starting position could put the maximum remnant flux density at the lowest possible value, approximately 60% of the maximum density, so that saturation would be least likely to occur. However, the “separated loop” shielding coils with a 0° starting position featured the lowest heating value, approximately 50% of the maximum value, and thus had the greatest potential to reduce the usage of copper for the shielding coils. For systems with other bus-bar layouts, the conclusion may be different, but the analysis procedure will be similar.

It should be noted that the empirical equations proposed in this paper only apply to the mainstream CTs within the dimensional range defined in Table 1, and to coils with rectangular cross sections as illustrated in Figure 2b. For coils with rounded corners, the core-pickup factor might be slightly different. Future work may focus on the physical essence of the core-pickup factor, for which a wider application scope of the analytical models can be expected.

## Figures and Tables

**Table 1:** Typical dimensional range of the mainstream CT.

Part	Parameter	Range
Ring core	*b*	0.20–0.45 m
	(*b − a*)/*b*	0.02–0.07 m
	*h*	0.02–0.06 m
Adjacent bus-bar	*c*	0.60–2.00 m

**Table 2:** Simulation cases for analysis on the core-pickup factor (stray flux).

Case	*r* (m)	*w* (m)	*h* (m)	*c* (m)
Case 1	0.19–0.44	0.035	0.025	1.2
Case 2	0.40	0.03–0.07	0.025	1.2
Case 3	0.40	0.035	0.02–0.07	1.2
Case 4	0.40	0.035	0.025	0.9–1.5

**Table 3:** Coefficients for the core-pickup factor equation (stray flux).

Coefficient	Value	Coefficient	Value
*p*_1_	1.4316	*p5*	−9.3438
*p*_2_	0.3194	*p6*	4.5496
*p*_3_	−0.2300	*p7*	3.2412
*p*_4_	−0.0453	*p8*	−0.3716

**Table 4:** Parameters of simple CT.

Part	Parameter	30 kA CT	25 kA CT	5 kA CT
Ring core	Inner radius *a*	0.38 m	0.37 m	0.1775 m
	Outer radius *b*	0.415 m	0.42 m	0.215 m
	High *h*	0.025 m	0.06 m	0.02 m
Coil	Winding angle α	120°	120°	120°
	Coil gap *d*_c_	0.05 m	0.05 m	0.05 m
	Coil thickness *t*_c_	0.01 m	0.01 m	0.01 m

**Table 5:** Parameters of the 30 kA CT Sample.

Part	Parameter	Range
Ring core	Inner radius *a*	0.380 m
	Outer radius *b*	0.415 m
	Height *h*	0.025 m
Bus-bars	Rated current *I*_0_ (RMS)	30 kA
Shielding coils	Gap between coil and core *d*_c_	1.200 m
	Thickness of coil *t*_c_	0.01 m
	Number of turns of each segment	686
	Inner resistance	0.70 Ω

**Table 6:** Experimental Results of the Shielding Coil Current.

Coil	Analytical	Simulation	Experimental
1–1′	2.554 A	2.511 A	2.085 A
2–2′	2.554 A	2.511 A	2.242 A
3–3′	2.554 A	2.511 A	2.672 A
4–4′	2.554 A	2.511 A	2.831 A
Average	2.554 A	2.511 A	2.458 A

**Table 7:** Parameters of the 30 kA CT Sample.

Part	Parameter	Range
Ring core	Inner radius *a*	0.1775 m
	Outer radius *b*	0.215 m
	Height *h*	0.02 m
Secondary winding	Number of turns	1000
	Internal resistance	1.0 Ω
Bus-bars	Rated current *I*_0_ (RMS)	5 kA
	Adjacent distance	0.70 m
Shielding coils	Gap between coil and core *d*_c_	0.03 m
	Thickness of coil *t*_c_	0.01 m
	Number of turns of each segment	337
	Inner resistance	0.35 Ω

**Table 8:** Experimental Results of the CT Performance Parameters.

Topology	*β*	|*Φ*_r_|_max_ Analytical	|*Φ*_r_|_max_ Experimental	$I_{b-\max}^{2}$ Analytical	$I_{b-\max}^{2}$ Experimental
		(10^−5^ Wb)	(10^−5^ Wb)	(A^2^)	(A^2^)
All parallel	0°	4.480	5.605	1.063	1.742
	45°	4.083	5.495	1.096	1.750
Separated loop	0°	5.114	6.120	0.659	1.300
	45°	4.083	5.483	1.096	1.610
